# Sex Differences in Metabolic and Behavioral Responses to Exercise but Not Exogenous Osteocalcin Treatment in Mice Fed a High Fat Diet

**DOI:** 10.3389/fphys.2022.831056

**Published:** 2022-03-02

**Authors:** Jordan Winberg, Jesse Rentz, Kim Sugamori, Walter Swardfager, Jane Mitchell

**Affiliations:** ^1^Department of Pharmacology and Toxicology, University of Toronto, Toronto, ON, Canada; ^2^Sunnybrook Research Institute, Toronto, ON, Canada

**Keywords:** exercise, osteocalcin, Type 2 diabetes, depression, anxiety, cognition

## Abstract

**Background:**

Exercise helps improve glucose handling in diabetes and has been shown to improve mood and cognition in other conditions. Osteocalcin, a protein produced by bone osteoblasts, was reported to have endocrine actions to improve both metabolism and also improve age-related cognitive deficits in mice.

**Methods:**

This study was designed to compare the effects of daily treadmill running exercise with injection of osteocalcin in high fat diet (HFD) induced diabetes in male and female C57BL/6J mice. Following established glucose intolerance and treatment for 8 weeks, mice were assessed for anxiety on an elevated plus maze, motivation by tail suspension test and cognition and memory in a puzzle box. Endogenous osteocalcin was measured by ELISA.

**Results:**

Mice on HFD had high weight gain, glucose intolerance and increased white fat. Exercise increased circulating osteocalcin levels in female mice but decreased them in male mice. Exercise also decreased weight gain and improved glucose tolerance in female but not male mice; however, treatment with osteocalcin made no metabolic improvements in either males or females. HFD induced anxiety only in female mice and this was not improved by osteocalcin. Exercise induced anxiety only in male mice. HFD also increased depressive-like behavior in both sexes, and this was improved by either exercise or osteocalcin treatment. Cognitive deficits were seen in both male and female mice on HFD. Exercise improved cognitive performance in female but not male mice, while osteocalcin treatment improved cognitive performance in both sexes.

**Conclusion:**

There were sex differences in the effects of exercise on endogenous osteocalcin regulation that correlated with improvements in cognitive but not metabolic outcomes. Exogenous osteocalcin did not improve metabolism but was effective in improving HFD-induced cognitive deficits. Sex is an important variable in hormonal and cognitive responses to exercise in diabetes.

## Highlights

- A high-fat diet induced similar metabolic changes in male and female mice.
- Female mice on high-fat diet were more anxious, while male mice became anxious on a high-fat diet only if they were exercised.
- Female mice benefited metabolically and cognitively from daily exercise more than male mice.
- Administration of exogenous osteocalcin affected behavior and cognition but not metabolism.
- There were sex differences in the effects of exercise on endogenous osteocalcin in this T2DM mouse model.

## Background

Type 2 diabetes mellitus (T2DM) is a highly prevalent condition accounting for 90% of all diabetes mellitus cases (International Diabetes Federation, 2019). T2DM is characterized by impaired insulin sensitivity, hyperinsulinemia, and hyperglycemia (DeFronzo et al., 2015). In addition to higher risks of cardiovascular, renal and retinal disease, recent studies indicate people with T2DM have increased risk of developing depression, anxiety, and cognitive impairment (Anderson et al., 2002; Semenkovich et al., 2015; van Gemert et al., 2018).

Interventions for treating T2DM commonly include both changes to lifestyle and medication to improve glycemic control. Exercise promotes weight loss, improves glycemic control and cardiovascular health (Swoboda et al., 2016; Tomas-Carus et al., 2016; Chiang et al., 2019). Increasing exercise can improve glycemic control, increase insulin sensitivity, glucose uptake and utilization (Hawley and Lessard, 2007; Magkos et al., 2008). Furthermore, exercise has beneficial impacts on the brain (Raichlen and Alexander, 2017). Mice also benefit from regular exercise. Guo et al. (2020) reviewed over 300 studies investigating the effects of exercise in mice and found that regardless of exercise type (i.e., treadmill, wheel running, swimming), a majority of animals demonstrated positive effects in models of neurodegenerative and metabolic disorders. The physiological mechanisms underlying many of the benefits of exercise on physical and mental health remain unclear.

The osteoblast-derived peptide, osteocalcin, is the most abundant non-collagenous protein in the bone matrix (Hauschka et al., 1989). The mature form of human osteocalcin undergoes vitamin K-dependent carboxylation at glutamate residues 17, 21, and 24 (Hauschka et al., 1989). Osteocalcin also circulates in the blood in uncarboxylated and a variety of partially carboxylated states. The uncarboxylated form of osteocalcin is suggested to be bioactive (Lee et al., 2007). Several studies have suggested roles for osteocalcin in regulating metabolism, behavior and cognition based on a mouse model lacking osteocalcin (Lee et al., 2007; Oury et al., 2013), and further studies found that daily injections of exogenous osteocalcin decreased the severity of diet-induced obesity and glycemic control in a mouse model of T2DM (Ferron et al., 2008, 2012). However, two other mouse lines lacking osteocalcin have recently been reported to have no endocrine phenotype (Diegel et al., 2020; Moriishi et al., 2020). Further studies have shown osteocalcin can cross the blood-brain barrier to improve cognition and alleviate anxiety and depressive-like behaviors in older mice (Oury et al., 2013; Khrimian et al., 2017).

The effects of osteocalcin in mice appear to be similar to the metabolic and cognitive effects of regular exercise; however, it is unclear if exercise stimulates the release of osteocalcin from bone. Some studies have shown exercise increases serum osteocalcin concentrations in humans, mice and rhesus monkeys (Levinger et al., 2011; Kim et al., 2015; Mera et al., 2016), while others have shown no changes in osteocalcin in response to acute exercise in humans (Welsh et al., 1997; Herrmann et al., 2007). Yet another study has shown sex differences in osteocalcin in response to exercise where osteocalcin decreased in men, but not in women, 1 day following distance running (Brahm et al., 1996), raising the possibility of sex differences in the neuroendocrine effects of physical exercise.

The experiments in this study were designed to compare the effects of exercise and osteocalcin administration in male and female mice using the well-characterized, high fat diet-induced mouse model of T2DM. We show that there are a number of sex differences in response to these two interventions.

## Materials and Methods

### Animals and Treatments

Studies in male and female mice were conducted with C57BL/6J mice from Jackson Labs (Bar Harbor, ME, United States) beginning at 4-weeks of age and housed under standard conditions with a 12-h light-dark cycle, and water and food *ad libitum*. Mice were fed either a high-fat diet (HFD) that provided 60.3% of calories from fat, 21.4% carbohydrate, and 18.3% protein (TD.06414, Envigo, Mississauga, ON, Canada) and compared to control mice (Control) fed a diet that provided 10.5% of calories from fat, 69.1% carbohydrate and 20.5% from protein (Envigo, TD.08806). The mice were housed in groups of 3–4 and fed their respective diets for 8 weeks. At 12 weeks of age, mice were separated into individual cages, the control animals remained sedentary and received daily injections of phosphate buffered saline (PBS) and the HFD-fed mice were randomized into three groups (*n* = 9–12 per group) (1) sedentary receiving PBS injections, (2) exercise receiving PBS, or (3) sedentary receiving osteocalcin injections.

Prior to the beginning of exercise, mice in the exercised group were acclimatized to the 6-lane treadmill (Columbus Instrument, Columbus, OH, United States) at 8 m/min. During the 8 weeks of treatment, the mice were exercised 5 days per week between 8 and 11 a.m. Mice were placed on the treadmill and allowed to explore prior to warm up periods at 4 and 8 m/min for 2 mins each, followed by exercise session at 12 m/min for 30 min. We have previously used this exercise regimen in FVB male mice to increase bone formation (Dela Cruz et al., 2016).

For treatment with osteocalcin, lyophilized uncarboxylated Glu-osteocalcin (AnaSpec, AS-65307-025, Freemont, CA, United States) was dissolved in PBS to a stock concentration of 300 ng/μL and stored in aliquots at −80°C. Fresh aliquots were diluted in PBS to a working concentration of 12 ng/μL each day of treatment. Mice received 30 ng/g of Glu-osteocalcin subcutaneously or equivalent volume of PBS each day at 2 p.m. The dose of 30 ng/g of Glu-osteocalcin was previously demonstrated as sufficient to reduce the severity of HFD induced T2DM (Ferron et al., 2012).

### Metabolic Assessments

Animal weights and food weights were recorded weekly to track weight gain and food consumption. The percent change in bodyweight was calculated as the change in weight from the first to the last week of the 8-week treatment period.

Glucose tolerance tests were performed to determine the effects of treatment on metabolic status. Mice were fasted for 6 h before assessment of blood glucose levels in a drop of blood from the tail vein using a glucometer (Contour Next, Ascensia Diabetes Care, Mississauga, ON, Canada). Mice were injected i.p. with 2 mg/kg bodyweight of glucose in PBS. Blood glucose was measured at 15, 30, 60, and 120 mins following glucose injection. The concentration range of the glucometer was 0.6–33.3 mM and mice with glucose levels above the maximum were reported as 33.3 mM. The fasting blood glucose and area under the curve (AUC) were used as measures of glucose tolerance.

### Behavioral Tests

During the final 2 weeks of treatments animals were subjected to one test per day with 24 h interval between tests. The open field test (OFT; Omnitech Electronics Inc., Columbus, OH, United States) was performed as described (Seibenhener and Wooten, 2015) to assess the impact of diet and treatments on locomotor activity. The OFT chamber (42 cm × 42 cm) was subdivided into quadrants (20 cm × 20 cm) to enable analysis of two mice simultaneously in opposite corners. Light intensity of the room was adjusted to achieve 50 lux inside the chamber. Mice were placed in the center of the quadrant and allowed to explore for 30 mins. Animals were tracked by video camera and recorded using Fusion Software (Version 4.7.1.4, Omnitech Electronics, Columbus, OH, United States). Distance traveled and time spent moving were used as measures of locomotor activity. Additional measures of time spent in the center vs periphery of the field as an indicator of anxiety were not measured because the confined field area of the quadrant made these measures invalid.

To assess anxiety-like behaviors in our mice, the elevated plus maze was performed as described (Komada et al., 2008). Briefly, mice were placed in the center zone facing a closed arm and were allowed to explore for 10 mins. Animals were tracked by video camera and analyzed using Biobserve Viewer 3 software (Biobserve, Bonn, Germany). The time spent in the open arms was calculated as the percent of the total time in both open and closed arms [time open/(time open + closed) × 100], and entry ratio was calculated as the ratio of entries to the open arms to closed arm entries (open entry/closed entry × 100).

To assess motivation to escape, the tail suspension test was performed to assess depressive-like behaviors in the mice (Steru et al., 1985). The proximal end of the tails of mice were covered with a plastic tube to dissuade mice from climbing up their tails as they were suspended 20 cm above a padded base. Each animal was suspended for 6 min and the time that the animal spent attempting to escape was recorded as mobile time. Percent mobile time was calculated as time spent moving divided by total test time.

The puzzle box test was adapted from previously published method to assess cognitive function (Ben Abdallah et al., 2011). The test was conducted in a standard light-dark box. The test took place over 3 days with three trials per day separated by 2 min intervals in the home cages between trials. For each stage of the test, the mice were placed in the light compartment of the box and allowed to explore for a maximum of 5 min. Mice that failed to complete the challenge within 5 min were removed from the arena and a time of 5 min was recorded for the attempt. Time to completely enter the dark compartment was measured as latency to solve the challenge. On the first day, mice were placed in the box with an open door to the dark compartment. The next two challenges had the door closed, with entrance through an underpass. On the second day, the mice repeated the closed-door challenge followed by two challenges with the underpass filled with bedding. The bedding challenge was repeated at the start of the third day followed by two challenges with a cardboard plug inserted into the underpass. Problem-solving was used as a measure of cognitive function and was calculated as the latency to solve each of the challenges. A combined problem-solving score was calculated by summation of Z-scores for latency to solve each of the increasingly difficult tasks on the first attempt.

### Tissue Collection and Osteocalcin Assays

Animals were euthanized by a combination of isoflurane overdose and cardiac puncture. Visceral fat was collected from around the gonads and inguinal fat was collected as a measure of sub-cutaneous fat. Extracted fat pad weights were expressed as a percentage of bodyweight at the time of collection.

Blood collected at the time of euthanasia was left at room temperature to clot then centrifuged at 1,750 RPM in a microfuge for 15 min to collect serum. Serum mouse osteocalcin concentrations were determined using ELISAs specific to mouse carboxylated osteocalcin (Gla-OC MK127, Takara Bio United States, Mountain View, CA, United States) and uncarboxylated osteocalcin (Glu-OC MK129, Takara Bio).

### Statistical Analysis

Figures were generated using Graphpad Prism 5.0 Software. Statistical analyses were carried out using R (version 3.6.1). Behavioral data were Z-normalized to the male controls. Z-scores were calculated by subtracting the mean measure of the male control group from each value and dividing the difference by the standard deviation of the male control group. Student *t*-tests were used for simple comparisons between groups with Bonferroni adjustment for multiple comparisons (*p* = 0.05/number of comparisons) significant differences are indicated by * and *p* values are shown above the bars in each figure. Three-way ANOVAs were used to assess the impact of sex, exercise and osteocalcin and to explore for interactions (sex × exercise and sex × osteocalcin). Data are presented as the mean ± standard deviation.

## Results

### Metabolic Outcomes

**FIGURE 1:**
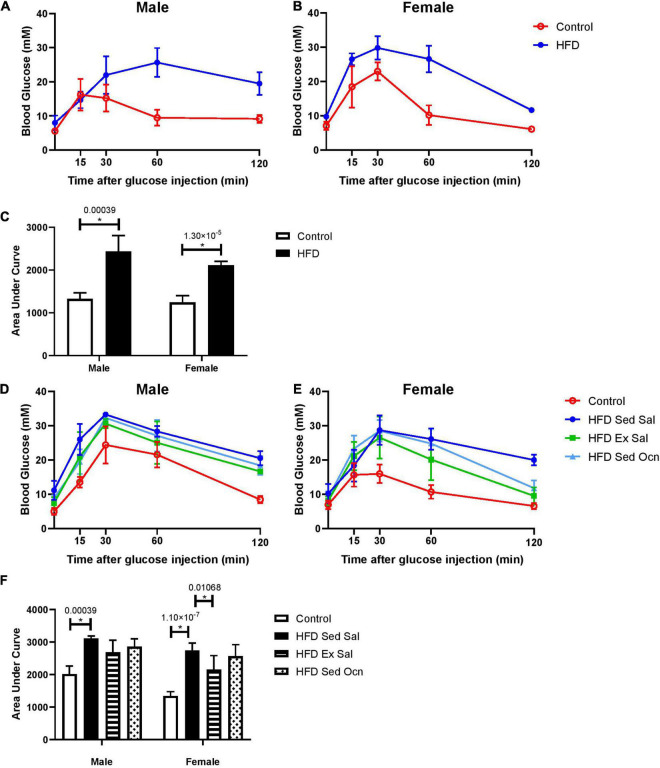
Glucose tolerance tests. At 12 weeks of age after 8 weeks of control diet (Control) or high fat diet (HFD); **(A)** male, **(B)** female time-courses, **(C)** area under the curve. Mice at 20 weeks of age following 8 weeks of treatments **(D–F)**, sedentary mice on control diet with daily saline injection (Control), sedentary mice on HFD (HFD Sed Sal), exercised mice on HFD (HFD Ex Sal) or sedentary mice on HFD treated with osteocalcin (HFD Sed Ocn). **(D)** Male mice, **(E)** female time-courses, and **(F)** area under the curve. Error bars represent standard deviation from the mean; 8-week GTT male groups *n* = 6, female groups *n* = 5; end of treatment GTT Male groups *n* = 5, female groups *n* = 7. *Indicates significant differences after Bonferroni correction, unadjusted *p* values are shown above bars.

After 8-weeks on the HFD, before commencement of treatments, both male and female mice showed significantly impaired glucose tolerance compared to mice on the control diet (Figures 1A–C). Following the treatment period, HFD-fed males and females continued to have impaired glucose tolerance; however, exercised females had significantly improved glucose tolerance (Figures 1D–F). As well, while body weights were significantly higher in HFD-fed animals, exercised females displayed significantly less weight gain than HFD-fed sedentary males (Table 1). Exercise improved glucose tolerance and decreased weight gain in females but not in males. Daily injection with osteocalcin had no apparent metabolic effects in males or females (Figure 1E and Table 1). Average daily caloric intake normalized to body weight was not significantly affected by diet, exercise, or injection with osteocalcin in males or females (Table 1).

**TABLE 1:** Metabolic measures after 8 weeks of treatments.

	Male				Female			
	Control	HFD Sed Sal	HFD Ex Sal	HFD Sed Ocn	Control	HFD Sed Sal	HFD Ex Sal	HFD Sed Ocn
Body weight (g)	27.01 ± 1.25	41.73 ± 3.50***	40.67 ± 3.35	45.78 ± 4.09	21.47 ± 1.52	31.23 ± 3.81***	27.82 ± 3.35	30.30 ± 3.60
Percent change in weight (%)	8.457 ± 5.407	28.21 ± 16.41*	20.83 ± 9.75	28.66 ± 5.38	3.583 ± 5.842	15.44 ± 6.453**	3.162 ± 8.783^##^	12.78 ± 8.388
Caloric intake per BW per day	0.365 ± 0.013	0.361 ± 0.035	0.380 ± 0.043	0.363 ± 0.048	0.425 ± 0.029	0.402 ± 0.034	0.414 ± 0.082	0.409 ± 0.051
Subcutaneous fat (% of body weight)	0.720 ± 0.147	2.296 ± 0.600**	1.777 ± 0.609	2.732 ± 0.474	2.187 ± 0.712	5.753 ± 1.133***	4.927 ± 1.680	5.074 ± 1.396
Visceral fat (% of body weight)	2.911 ± 0.176	7.149 ± 1.033***	6.820 ± 0.620	5.709 ± 1.042^[*dollar*]^	2.113 ± 0.631	5.649 ± 0.942***	4.874 ± 1.798	5.332 ± 1.673
Gla-osteocalcin (ng/mL)	36.76 ± 5.67	33.21 ± 5.24	23.38 ± 7.48^#^	31.54 ± 4.50	47.46 ± 15.90	51.85 ± 14.60	79.15 ± 23.73^#^	49.18 ± 12.33
Glu-osteocalcin (ng/mL)	1.38 ± 0.29	1.12 ± 0.29	0.72 ± 0.21^#^	0.98 ± 0.21	1.53 ± 0.53	1.69 ± 0.58	2.12 ± 0.49	1.69 ± 0.43

Values represent means ± SD. HFD Sed Sal to Control: *p < 0.0167; **p < 0.001; ***p < 0.0001; HFD Ex Sal to HFD Sed Sal: #p < 0.0167; ##p < 0.001. HFD Sed Ocn to HFD Sed Sal: $p < 0.0167; p-values < 0.0167 were considered significant after Bonferroni correction.

The HFD significantly increased the accumulation of both subcutaneous and visceral fat in males and females (Table 1). All of the female groups had significantly greater subcutaneous fat than their respective male groups (Table 2). Male animals on HFD given osteocalcin had significantly less accumulation of visceral fat than those that received saline (Table 1). Daily exercise had no apparent effects on fat accumulation in HFD-fed male or female mice.

**TABLE 2:** Summary of ANOVA outputs.

Measure	Sex	Exercise	Osteocalcin	S × E	S × O
Body weight	*F*(1, 57) = 197.02, *p* < 0.001	*F*(1, 57) = 9.64, *p* < 0.01			*F*(1,57) = 4.86, *p* < 0.05
Percent change in weight	*F*(1,57) = 40.10, *p* < 0.001	*F*(1,57) = 13.82, *p* < 0.001			
Caloric intake per BW per day	*F*(1,57) = 9.25, *p* < 0.01				
GTT AUC	*F*(1,30) = 14.23, *p* < 0.001	*F*(1,30) = 14.09, *p* < 0.001			
Percent visceral fat	*F*(1,56) = 61.21, *p* < 0.001				
Percent subcutaneous fat	*F*(1,56) = 221.54, *p* < 0.001				
Gla-OCN	*F*(1,42) = 62.86, *p* < 0.001	*F*(1,42) = 7.26, *p* < 0.05		*F*(1,42) = 19.69, *p* < 0.001	
Glu-OCN	*F*(1,42) = 61.64, *p* < 0.001			*F*(1,42) = 9.86, *p* < 0.01	
OFT movement time	*F*(1,57) = 5.73, *p* < 0.05				
OFT distance	*F*(1,57) = 38.40, *p* < 0.001	*F*(1,57) = 11.72, *p* < 0.01			
EPM% open arms		*F*(1,54) = 4.61, *p* < 0.05			
EPM arms entry ratio		*F*(1,54) = 7.56, *p* < 0.01			
TST mobile time		*F*(1,57) = 14.59, *p* < 0.001	*F*(1,57) = 55.69, *p* < 0.001		
Puzzle box problem-solving	*F*(1,57) = 28.30, *p* < 0.001		*F*(1,57) = 52.00, *p* < 0.001	*F*(1,57) = 15.73, *p* < 0.001	

### Serum Osteocalcin

The HFD had no effect on circulating endogenous osteocalcin concentrations in either male or female mice (Table 1). All HFD-fed female groups had significantly higher levels of serum osteocalcin than their male counterparts. Exercised males had significantly reduced serum carboxylated Gla-osteocalcin and uncarboxylated Glu-osteocalcin relative to the sedentary animals, whereas exercised females had significantly increased serum Gla-osteocalcin and a trend to higher levels of Glu-osteocalcin, relative to the female sedentary group (Table 1); the interaction between sex and exercise for both serum Gla- and Glu-osteocalcin levels was significant in a three-way ANOVA model (Table 2).

### Behavioral Outcomes

#### Basal Mobility

**FIGURE 2:**
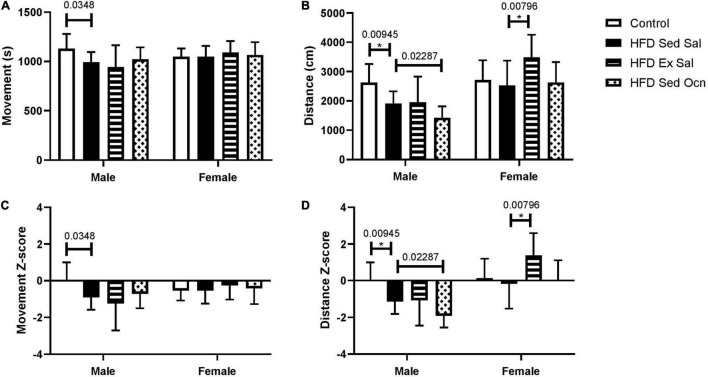
Open field tests. **(A)** Movement time represents the time spent moving (s) and **(B)** distance represents the length of the path traveled (cm). **(C)** Movement time Z-normalized to the male mice on control diet (Control). **(D)** Distance traveled Z-normalized to the male mice on control diet (Control). Bars represent means with standard deviation shown in the error bars; male control group *n* = 10, HFD groups *n* = 9; female control group *n* = 10, HFD groups *n* = 12, *Indicates significant differences after Bonferroni correction, unadjusted *p* values are shown above bars.

In the open field test in the final week of treatment, the HFD had no significant effect on movement time in males or females (Figure 2). All HFD-fed female groups traveled significantly more in the open field test than their respective male groups; ANOVA indicated a significant effect of sex on both movement time and distance traveled (Table 2). The HFD decreased the distance traveled in males but not in females. Exercise significantly increased the distance traveled by females but not males. Osteocalcin treatment had no apparent effects on locomotion in males or females.

#### Anxiety-Like Behavior

**FIGURE 3:**
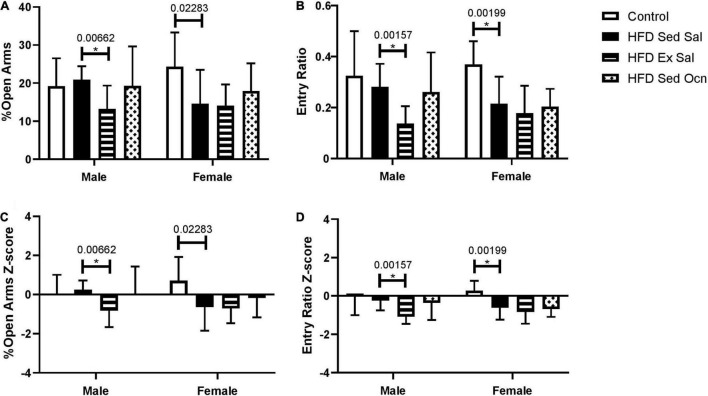
Elevated plus maze. **(A)** Percent of total time exploring in the open arms. **(B)** The ratio of entries to open arms to entries to closed arms. **(C)** Z-normalized to the male control group for percent of total time exploring in the open arms. **(D)** Z-normalized to the male control group for ratio of entries into open and closed arms. Bars represent means ± standard deviation; male control group *n* = 10, HFD groups *n* = 9; female control group m = 10, HFD groups *n* = 11. Statistically significant differences after Bonferroni correction, indicated by * with unadjusted *p* values shown above bars.

In an elevated plus maze, female mice on a HFD had increased anxiety-like behaviors but males did not. Exercise significantly increased anxiety-like behaviors in HFD-fed males, as shown by decreased percentage of time spent in the open arms and a decreased ratio of visits to the open arms of the maze (Figure 3). Exercise did not significantly change anxiety-like behaviors in females; however, ANOVA indicated a significant effect of exercise on both percent of time spent in the open arms and open arm entry ratio overall considering both sexes (Table 2).

#### Depressive-Like Behavior

**FIGURE 4:**
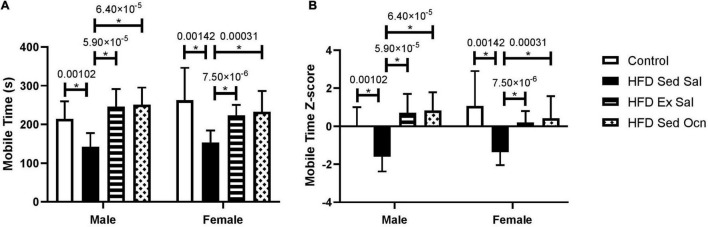
Tail suspension test. **(A)** Time (s) spent mobile during a 6 min tail-suspension test. **(B)** Z-normalized data to the male control group. Bars represent mean values ± standard deviation; male control group *n* = 10, HFD Sed Sal *n* = 10, HFD Ex Sal *n* = 9, HFD Sed Ocn *n* = 8; female control group *n* = 11, HFD groups *n* = 12. Statistically significant differences after Bonferroni correction indicated by * with unadjusted *p* values shown above bars.

In the tail suspension test, the HFD significantly increased depressive-like behavior in both males and females based on decreased mobility time (Figure 4). Immobility time during a 6-min tail suspension test was not different between males and females. In both males and females, exercise treatment and osteocalcin treatment significantly reduced this depressive-like behavior; in ANOVA, both exercise and osteocalcin treatments, but not sex, had significant effects on depressive-like behavior (Table 2).

#### Problem-Solving

**FIGURE 5:**
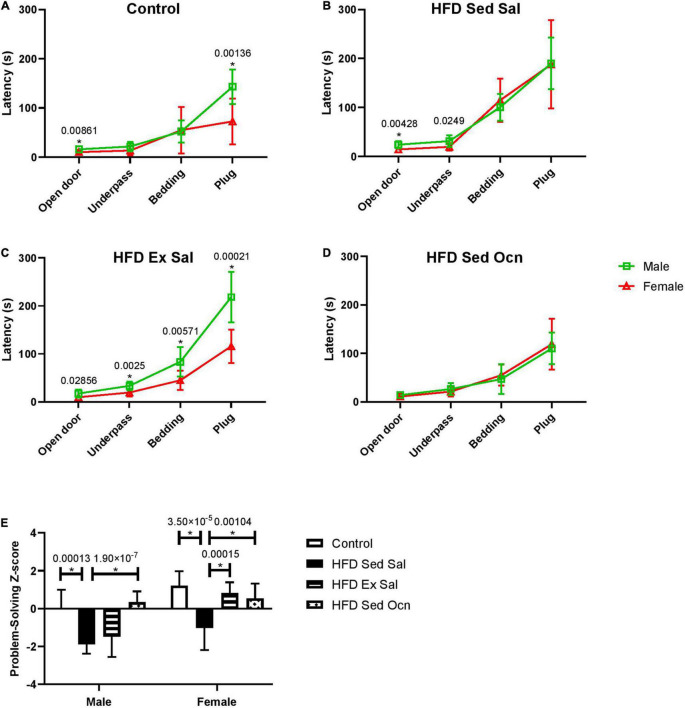
Progressive problem-solving in puzzle box. Latencies for males and females of each treatment group during the puzzle box test **(A–D)** squares represent data from males, triangles represent data from females. **(A)** Controls; **(B)** High fat diet, sedentary with daily saline injections (HFD Sed Sal); **(C)** High fat diet, daily exercise and saline injections (HFD Ex Sal); **(D)** High fat diet sedentary with daily osteocalcin injections (HFD Sed Ocn); **(E)** Problem-solving scores, Z-normalized to the male control group. Data point and bars represent mean ± standard deviation; male control *n* = 10, HFD groups *n* = 9; female control *n* = 10, HFD *n* = 12. Statistically significant differences after Bonferroni correction indicated by * with unadjusted *p* values shown above bars.

In the puzzle box test of problem-solving ability, performance on consecutive tasks with increasing difficulty over 3 days (Figures 5A–D) was aggregated into a single Z-score, calculated relative to the male control group, with positive scores indicative of faster completion times (better performance) and negative scores indicative of slower completion times (Figure 5E). The HFD significantly decreased puzzle box performance compared to the control diet for both males and females (Figure 5E). Exercise only improved problem-solving scores in females; ANOVA indicated a significant interaction between sex and exercise (Table 2). Exercised females performed significantly faster than exercised males on each task (Figure 5C). Osteocalcin treatment significantly improved problem-solving scores in both males and females and there was no interaction between osteocalcin and sex (Table 2). Osteocalcin treated males and females had similar latencies to complete each task (Figure 5D). A combined problem-solving score was generated by summing the Z-scores for latency to solve each of the increasingly difficult tasks (open door, underpass, bedding, and plug) on the first attempt and again Z-normalizing to the male control group (Figure 5E). Female mice were significantly faster overall at solving the tasks than males and exercise improved the problem-solving score only in the female mice, while osteocalcin treatment improved the scores of both male and female mice (Figure 5E).

## Discussion

In this study we aimed to examine the role of sex in the metabolic and behavioral effects of a HFD-induced mouse model of T2DM, and to compare the effects of daily exercise with those of daily injection with uncarboxylated osteocalcin on the metabolic and behavioral effects of the HFD.

Within 8 weeks, the HFD caused weight gain and the development of glucose intolerance in both male and female mice, establishing a diabetic phenotype. The additional 8 weeks of HFD during the treatment period maintained the diabetic phenotype with continued weight gain that was far greater in males than females. Similarly, male mice on the HFD showed worse glucose tolerance than did the females, suggesting that metabolism in male mice is more affected by dietary fat levels than in females as previously reported (Gelineau et al., 2017). We observed a sex difference in an open field test that the HFD decreased distance traveled in male mice but not in females, perhaps contributing to the difference in weight gain between the sexes. Previous work has reported decreased locomotion in male mice fed a high-fat Western diet after as little as 3–5 h on diet (Bjursell et al., 2008) and Gelineau et al. (2017) also showed that locomotor activity decreased in male C57BL/6 mice fed a HFD, but not in females.

Treadmill running for 8 weeks was used as a potential method of improving metabolic health in this study. Exercise only decreased weight gain and improved glucose tolerance in female mice. Weight loss may have reflected an overall loss of body fat; however, this was not significant in the fat pads we measured. This suggests that a more rigorous exercise regimen may lead to even better weight and fat loss in these mice. Very few studies have compared effects of treatments for diabetes in both sexes of mice. Here we saw sex differences, including no metabolic improvements in male mice using the same exercise regimen as the females. Our results concur with those of others, which suggested previously that voluntary wheel running plays a more limited role in reducing obesity and diabetic symptoms in male mice when fed a HFD (Hatzidis et al., 2017).

Further differences in the effects of diet and exercise between male and female mice were seen when we examined their behavioral profiles. Only female mice showed HFD-induced anxiety as seen by the elevated plus maze and neither of the treatments alleviated this effect. In male mice we saw no effect of a diet high in fat from lard but in previous studies the type of fat in the diet was found to influence anxiogenic effect, with 4 weeks of a diet high in lard increasing anxiety but not a diet high in fish oil (Mizunoya et al., 2013). Another study of 16 weeks of HFD increased anxiety-like behavior in male C57BL/6 mice as measured by the time in the open field (Zemdegs et al., 2016). A notable difference between these two studies and ours is that the mice in the previous studies were group-housed, whereas mice in our study were individually housed during the treatment period. Group housing for male mice is contrary to their natural environment and known to increase stress levels in the animals (Kappel et al., 2017) and may have influenced the acquisition of anxiety in male mice in these other studies in comparison to ours. Group housing in female mice is not contrary to their natural environment but in laboratory animals has been shown to increase their corticosterone levels compared to singly housed mice, while not altering their anxiety as assessed by several measures (Arndt et al., 2009). Another study of the effects of social isolation compared to group housing in male and female C57BL/6J mice did not show any effects of housing on anxiety-like behavior in either sex as measured in EPM or light-dark box tests (Huang et al., 2017). Thus it seems likely that the HFD-induced anxiety in the female mice in our study was induced by the diet and not by the housing conditions.

Both male and female mice showed evidence of HFD-induced depressive-like behavior and decreased problem-solving ability. HFD-induced depressive-like behaviors with prolonged immobile times in the tail suspension test for mice of both sexes. It cannot be ruled out that the slower movement of male mice on HFD, as seen in the open field test (Figure 2B), could have contributed to the immobility in the tail suspension test seen in males; however, both exercise and osteocalcin treatment improved depressive-like behavior in the male mice without changing performance in the open field test, suggesting that exercise and osteocalcin more likely had an effect on motivation to escape than ability to move. HFD-induced depressive-like behaviors in C57BL/6 mice fed a HFD have been seen previously in male mice but they had not been reported in females (Arndt et al., 2009; Yamada et al., 2011). There is also previous evidence of HFD-impaired learning and memory in male mice in the Morris water maze, Y-maze, and novel object recognition paradigms (Park et al., 2010; Cordner and Tamashiro, 2015), but again no comparisons with female mice had been reported previously.

Exercise had efficacy in improving depressive-like behavior in both sexes in our study. Previous suggestions of a link between anxiety and decreased problem solving in mice (Salomons et al., 2012), pose a possible explanation for our observed sex differences in exercise effects on performance in the puzzle box test. In female mice, exercise improved problem solving while there was no such effect in males, possibly because of the induction of anxiety-like behavior by exercise in males. Taken together our results suggest female mice benefit more than males from exercise to ameliorate some behavioral deficits induced by the HFD. This agrees with reported aerobic exercise improving learning and increasing brain-derived neurotrophic factor (Bdnf) expression more in female than in male mice (Klein et al., 2016), and with improved performance in various mazes or novel object recognition tests in female mice fed a HFD when given access to a running wheel (Klein et al., 2016; Robison et al., 2018).

In our study, administration of uncarboxylated osteocalcin (ucOcn) following established glucose intolerance did not alter further weight gain nor improve measures of glucose tolerance in either sex. This result is in contrast to previous studies which only examined effects of osteocalcin administration in male mice fed HFD, where it was shown that intermittent injections of ucOcn decreased weight gain and improved glucose tolerance in C57BL/6J male mice (Ferron et al., 2012; Zhou et al., 2013). We did observe that injection with ucOcn significantly decreased visceral fat accumulation in male mice but this was not sufficient to diminish overall weight gain or improve glucose tolerance in these mice with an established diabetic state. In female mice there were no metabolic benefits from ucOcn administration.

Despite the lack of effects of exogenous ucOcn on weight gain and glucose tolerance in our study, administration of ucOcn had profound effects on some facets of behavior in the mice similar to previously reported effects in older male mice (Khrimian et al., 2017). There were decreased depressive-like behaviors and improved problem-solving performance in both sexes. In female mice, the improvements by ucOcn administration were on par with those seen with exercise, and exercise increased endogenous osteocalcin in female mice, suggesting possible overlap in the mechanisms of the two treatments. In male mice, on the other hand, exercise decreased endogenous osteocalcin levels suggesting no link between endogenous osteocalcin levels and improvements in depressive-like behavior in males. To our knowledge, sex differences in the release of endogenous osteocalcin by exercise has not been reported previously and the results shown here require further investigation to determine how osteocalcin is released from bone by exercise and whether this is regulated by sex hormones.

## Conclusion

In conclusion, we observed metabolic benefits of exercise only in female mice and no metabolic benefits of osteocalcin treatment in either sex. We showed that osteocalcin administration improved depressive-like behavior and problem-solving performance in both sexes. Exercise increased anxiety-like behavior in male mice, which may have prevented improvements in cognition and memory. There were sex differences in the effects of exercise on endogenous serum levels of osteocalcin that correlated with improvements in cognition.

## Data Availability Statement

The raw data supporting the conclusions of this article will be made available by the authors, without undue reservation.

## Ethics Statement

The animal study was reviewed and approved by University of Toronto.

## Author Contributions

JW, JR, and JM planned and performed the experiments, analyzed the data, and drafted the manuscript. KS planned and performed serum analysis. WS and JM conceived the study, supervised the experiments, and contributed to the final manuscript. All authors read and approved the final manuscript.

## Conflict of Interest

The authors declare that the research was conducted in the absence of any commercial or financial relationships that could be construed as a potential conflict of interest.

## Publisher’s Note

All claims expressed in this article are solely those of the authors and do not necessarily represent those of their affiliated organizations, or those of the publisher, the editors and the reviewers. Any product that may be evaluated in this article, or claim that may be made by its manufacturer, is not guaranteed or endorsed by the publisher.
